# A microscopic mechanism study of the effect of binary surfactants on the flotation of Wiser bituminous coal

**DOI:** 10.1038/s41598-024-65466-7

**Published:** 2024-06-24

**Authors:** Chun Zhang, Xianju Qian, Hailong Song, Jinzhang Jia

**Affiliations:** 1 College of Safety Science and Engineering, Liaoning Technical University, Fuxin, 123000 Liaoning China; 2 Key Laboratory of Thermal Dynamic Disaster Prevention and Control of Ministry of Education, Liaoning Technical University, Huludao, 125105 Liaoning China

**Keywords:** Surfactants, Coal molecular structure, Molecular dynamics, Density function theory, Flotation, Mineralogy, Coal

## Abstract

Investigating surfactant effects on the floatability of Wiser bituminous coal holds significant importance in improving coal cleanliness and utilization value. Using density functional theory and molecular dynamics simulation methods, this study constructed models of Wiser bituminous coal and examined the impact of different surfactants, including the anionic surfactant sodium dodecyl benzene sulfonate, the cationic surfactant hexadecyl trimethyl ammonium bromide (CTAB), and the non-ionic surfactant fatty alcohol ethoxylated ether. The focus was on investigating the charge distribution characteristics of these molecules and the modifying effect of binary surfactants on the hydrophobicity of bituminous coal. Results revealed that the maximum electrostatic potential was concentrated near oxygen/nitrogen/sulfur-containing functional groups like sulfonic acid groups, quaternary ammonium cations, ethylene oxide, hydroxyl groups, carboxyl groups, and sulfur bonds. These functional groups exhibited a propensity for accepting/delivering electrons to form hydrogen bonds. Among the surfactants tested, CTAB revealed the slightest difference in frontier orbital energy, measuring 3.187 eV, thereby demonstrating a superior trapping ability compared with the other two surfactants. Adsorption reactions within the system were determined to be spontaneous, with over 60% of the interaction force attributed to electrostatic forces. Moreover, the repulsive force magnitude with water molecules followed the trend: sulfonate group (2.20 Å) < ethylene oxide (2.43 Å) < quaternary ammonium cation (2.57 Å), indicating more excellent water repellency of CTAB. Findings showed that CTAE binary surfactants proved most effective in modifying the hydrophobicity of bituminous coal. This study offers valuable insights into reducing waste, pollution, and resource wastage.

## Introduction

China's resource characteristics of "rich in coal, poor in oil, and low in gas" have determined the current coal-based energy consumption structure. The coal industry is an important basic industry of China's national economy, which has been driving the development of China's industrialization and urbanization for a long time. Coal dust is a complex mixture of chemical composition and physicochemical properties^1–3^, containing more than 50 elements and their oxides. The content of respirable dust can reach 40–95%, and it can also adsorb lead, copper, zinc, manganese, nickel, and other toxic dusts^3–6^. Consequently, flotation separation of coal^7,8^ dust has become a widely employed method in coal processing, serving to achieve both coal purification and de-ashing.

The presence of a considerable number of oxygen-containing functional groups on the surface of coal, including hydroxyl, carboxyl, and carbonyl^9,10^, renders it challenging to adhere with oily traps (e.g., diesel fuel, kerosene), which consequently impairs the flotation efficiency of coal. The use of surfactants to enhance the hydrophobicity of coal during the flotation process is a promising approach to achieving optimal flotation results^11–13^. The action mechanism of surfactants is to form a glue group in solution^14^, thereby reducing the surface tension of coal. This reduces the interaction between coal, water, and air bubbles, and promotes the attachment of air bubbles and the flotation separation of coal. As demonstrated by relevant scholars, the combination of different surfactants has an antagonistic effect on the wettability of coal, which is conducive to the flotation of coal^15^.

The presence of hydrophilic and lipophilic groups affects the adsorption behavior of surfactants on the coal surface, thus affecting the hydrophobicity of the coal. Xia et al.^16^ found that oxygen-containing functional groups in the low-rank coals were responsible for the restricted adsorption of the active agent molecules. Huang et al.^17^ investigated the adsorption behavior of methylene blue (MB) on bituminous coals, where, due to the polar interactions, the hydrogen bonds were formed between the surfactant molecules and water molecules and thus, the water molecules' activity was limited. In bituminous coals, the formation of hydrogen bonds between the surfactant molecules and water molecules due to polar interactions limited the activity of the water molecules. He et al.^18^ demonstrated that polar interactions were the primary influencing variable in the adsorption process, as evidenced by the adsorption of NPEO10 molecules at the water-coal interface. Bai et al.^19^ employed the XPS method to analyze the adsorption mechanism of surfactants on the surface of anthracite, using anionic (SDBS) and cationic (STAC) surfactants. The adsorption process covered a significant number of coal surface functional groups, resulting in a notable change in the elemental content of the modified coal surface. However, in outdoor operations such as coal mines, the performance of surfactants may be affected by a variety of factors, including temperature, humidity, mineral content, and others. These complex factors may not be considered comprehensively in the current research process.

In the research process, it is necessary to consider not only the type of surfactant but also other factors such as temperature, pressure, coal particle size, structure, moisture content, etc. These factors affect the hydrophobicity of coal. Xu et al.^20^ used three anionic surfactants as the object of research and demonstrated that the magnitude of the surface tension is the cause of the influence of the length of the wetting time at low surfactant concentrations. Furthermore, they showed that the density of adsorption depends on the interaction and electrostatic force of the surfactant and the coal molecules. You et al.^21^ demonstrated that the contact angle of NP-4 in sub-bituminous coal exhibited a fluctuating trend as the concentration of NP-4 increased. This was accompanied by a corresponding change in the negative interaction energy, indicating that the adsorption process was spontaneous. Chang et al.^22^ demonstrated that the addition of surfactants altered the charge and adsorption properties of coal, with varying effects observed across different types of coals and coal particle sizes. They also observed that the charge and zeta potential of coal were altered when the concentration of surfactant exceeded the critical micelle concentration (CMC). Furthermore, they found that the adsorption of surfactant increased with the increase in temperature when the concentration of surfactant exceeded the CMC. Although researchers and scholars have made significant strides in understanding the impact of surfactants on coal modification, there are still significant gaps in the research process. A comprehensive assessment of the environmental impact of surfactants in coal mining, processing, and use, including biodegradability, ecotoxicity, and potential long-term effects, is essential to inform decision-making and ensure the sustainable development of the coal industry.

In recent years, numerous researchers and scholars have enhanced the hydrophobicity or hydrophilicity of coal through molecular simulation^23–25^ with varying concentrations of surfactants, thereby providing a scientific foundation for the utilization of surfactants in the processes of coal washing and coalbed methane extraction, among others. Zhang et al.^26^ investigated the effect of surfactants on low-rank coal flotation from the perspectives of wetting rate and surface tension. Their findings indicate that the mixing of anionic and cationic surfactants can reduce the mutual repulsion between cationic surfactant molecules and enhance their arrangement in the solution. This provides a novel direction for the study of the mechanism of multiple surfactants in low-rank coal flotation. Nie^27^ and Zhao^28^ compared the wettability and adsorption of single and composite surfactants from both macroscopic and microscopic perspectives to further explore the effects of key factors such as concentration, molecular weight, and ionic type of surfactants on coal modification. Meng et al.^29^ conducted a molecular simulation study investigating the compounding of two surfactants, SDBS and AEO3. Their findings indicated that the influence of nonionic surfactants resulted in more compact adsorption sites on coal molecules, and the resulting compounding was significantly more effective than a single system for water adsorption. The study emphasized the selection and application of environmentally friendly surfactants to reduce the impact on the environment of coal in the process of coal treatment. This was achieved by analyzing the surface charge of molecular models according to density functional theory, confirming nucleophilic and electrophilic regions, elaborating the adsorption mechanism of hydrogen bonding^30^, and explaining the chemical stability and reactivity of activated molecules by using the bond energy difference^31^.

The flotation ability of coal samples modified with medium/high-rank coals, such as bituminous coal, is currently a widely studied topic. However, the enhancement of coal flotation performance by the mixed solution of multivariate surfactants cannot be overlooked. In order to investigate the change characteristics of binary surfactants on the hydrophobicity of Wiser bituminous coal, we conducted an electrostatic surface and frontier orbital analysis of the coal and different types of surfactants based on the methods of density functional theory (DFT) and kinetic simulation (MD). This analysis allowed us to calculate the interaction energies, radial distributions, and diffusion characteristics of water molecules under different surfactant systems. The mechanism of interaction between surfactant and coal molecules has been revealed, and some important results have been achieved in improving the hydrophobicity of bituminous coal. These results provide a guide for the research and development of surfactant modification of coal, improving the efficiency and selectivity of flotation, and reducing unnecessary impurities.

## Simulation settings and materials

### Material selection

Coal is a complex, tense, inhomogeneous, cross-linked macromolecular structure. The irregularity of the surface morphology and pore space leads to various adsorption forms on the coal surface. Coal is mainly composed of functional groups such as aromatic rings, hydrocarbon radicals, hydroxyl groups, carboxyl groups, etc., and the difference lies in the number and type of functional groups in the side chains of coals with different degrees of metamorphism^32^. Coal has an amorphous molecular structure^33^, and over the years many models for bituminous coals have been discovered. The Wiser bituminous coal model (C_192_H_166_N_4_O_19_S_9_) proposed in the mid-1970s was chosen for construction^17,34,35^ and is considered to be a more comprehensive and reasonable model^36,37^.

Surfactants can alter the hydrophilic/hydrophobic properties of coal by modifying the surface tension of the solution, influencing the interaction force between water and coal in contact, and consequently modifying the surface contact angle between water and coal. Surfactants are classified into four categories: anionic, cationic, nonionic, and amphoteric. The electrostatic potential of the particle surface is altered in different ways according to the charge of the surfactant.

**Figure 1:**
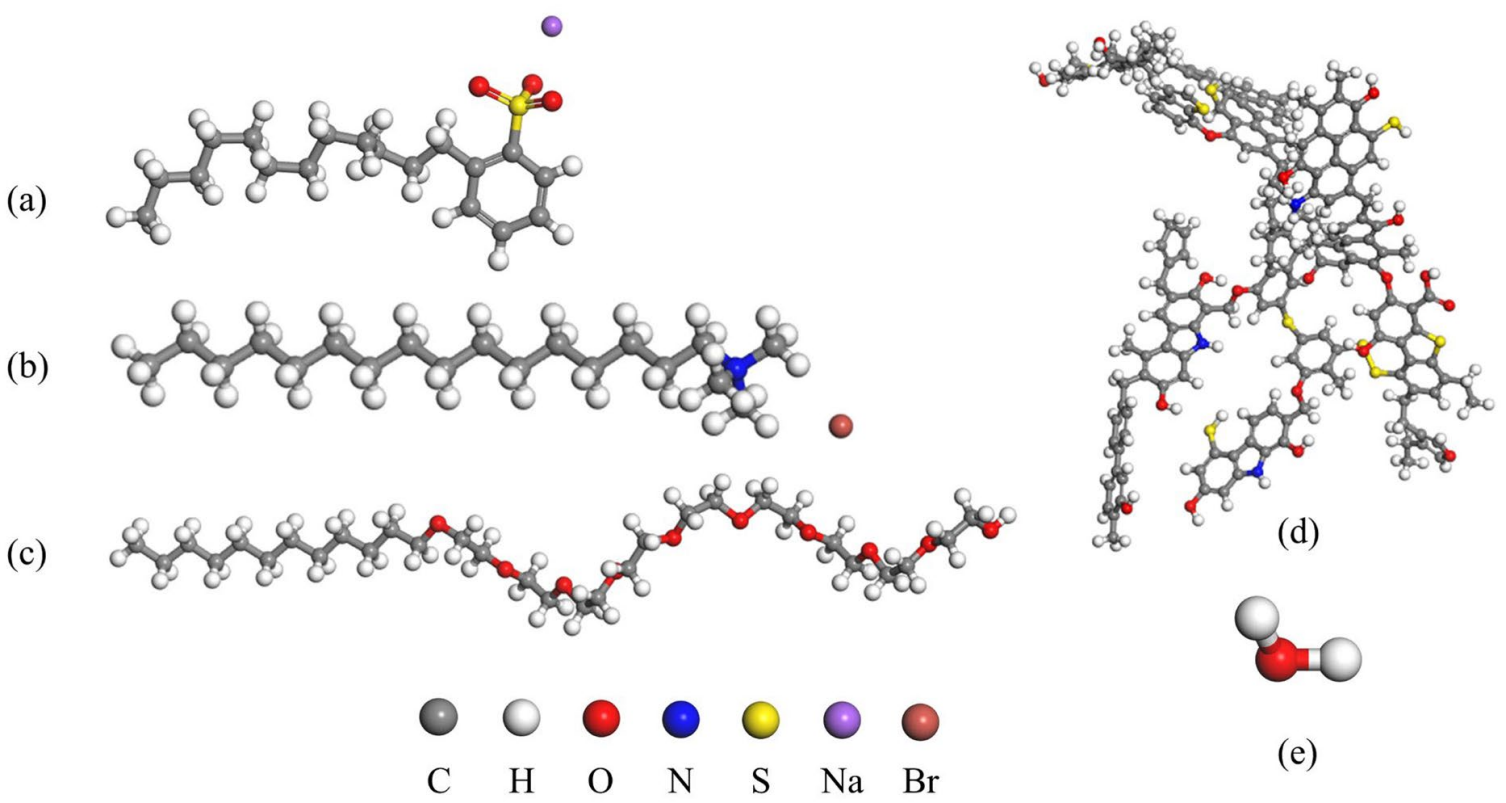
Molecular models of (**a**) Sodium dodecyl benzene sulfonate (SDBS), (**b**) Hexadecyl trimethyl ammonium bromide (CTAB), (**c**) Emulsifier AEO-9, (**d**) Wiser molecular model, (**e**) water.

Three surfactants were selected, including the anionic surfactant sodium dodecyl benzene sulphonate (SDBS), the cationic surfactant Hexadecyl trimethyl ammonium Bromide (CTAB) and the non-ionic surfactant fatty alcohol ethoxylated ether (AEO-9). The surfactant and carbon molecular models are shown in Fig. 1. The physical properties of the surfactants are given in Table 1.

**Table 1:** Physical properties of surfactants.

Surfactant	Molecular formula	Molecular weight	Density (g/cm^3^)	Critical micelle concentration (CMC) (mol/m^3^)
SDBS	C_18_H_29_NaO_3_S	348.48	1.02	1.5^38^
CTAB	C_19_H_42_BrN	364.46	1.32	0.90^39^
AEO9	C_30_H_62_O_10_	582.81	1.0 ± 0.01	0.10^39^

### Calculation method

#### Density functional theory (DFT)

Density functional theory (DFT) is a computational method to study the structure of multielectron architectures^40,41^, whose primary goal is to replace the wave function with the electron density as the fundamental quantity since the electron density is a function of three variables (x, y, z), the number of variables is much lower than that of the multielectron wave function with 3N variables (N is the number of electrons). Therefore, the arithmetic in the operation is greatly reduced. The electron density is calculated to represent the energy of the molecular system using DFT theory, and the formula is shown in (1).

1 $$\begin{aligned} E[\rho ] = & - \frac{{\hbar^{2} }}{{2m_{e} }}\sum\limits_{i = 1}^{n} {\int {\psi_{i}^{*} } } \left( {r_{i} } \right)\nabla_{1}^{2} \psi_{i} \left( {r_{i} } \right)dr_{1} - \sum\limits_{i = 1}^{N} {\frac{{Z_{1} e^{2} }}{{4\pi \varepsilon_{0} r_{I1} }}} \rho \left( {r_{1} } \right)dr_{1} \\ & + \frac{1}{2}\int {\frac{{\rho \left( {r_{1} } \right)\rho \left( {r_{2} } \right)e^{2} }}{{4\pi \varepsilon_{0} r_{12} }}} dr_{1} dr_{2} + E_{XC} [\rho ] \\ \end{aligned}$$

where *ρ*(*r*) is the electron density of the molecule, the first term to the right of the middle sign in the equation is the kinetic energy of the electrons, and the second term is the attraction potential between the nucleus and the electrons, the third term is the Coulombic interaction of the charges, and the last term is the exchange–correlation energy.

#### Electrostatic potential and frontier orbitals

The electrostatic potential is the amount of work required to move a unit of positive charge from a position at infinity to a position at a point in the space around the molecule. The electrostatic potential of the molecule is plotted using the Dmol3 module and shows the distribution of electrostatic potential values for a given charge density. This can be expressed using Eq. (2).

2 $$V(r) = \sum\limits_{A} {\frac{{Z_{A} }}{{\left| {R_{A} - r} \right|}}} - \int {\frac{{\rho \left( {r^{\prime } } \right)dr^{\prime } }}{{\left| {r^{\prime } - r} \right|}}}$$

Calculations of electron density and electrostatic potential are carried out by the Dmol3 module, and the electrostatic potential distribution diagram shows that the maximum and minimum electrostatic potentials are distributed in different regions, thus predicting the reaction sites where electrophilic and nucleophilic reactions occur^42^, and then analyzing the characteristics of the individual molecules and charge distributions, as well as the interactions^43^. The results of the calculations were converted into units in the form of the following Eq. (3).

3 $$\begin{aligned} 1a.u. = & Hartree = 27.21070eV \\ 1eV = & { 1}{\text{.602176565}} \times 10^{ - 19} J \\ \end{aligned}$$

In molecular orbitals, the highest occupied molecular orbital and the lowest unoccupied molecular orbital are collectively known as the front orbitals. The electrons in the front orbitals are like the valence electrons in the atomic orbitals and are the most active electrons in chemical reactions. The highest occupied orbital (HOMO) is the highest energy level in the occupied orbital; the higher the energy, the more likely it is to lose electrons; the lowest empty orbital (LUMO) is the lowest energy level in the empty orbital, the lower the energy of the electrons the more likely it is to accept electrons^44^. These two types of orbitals are the most easily affected in molecular action, and they determine the electron transfer and energy gain and loss of the molecule.

#### Relative concentration distribution

The Relative Concentration (*RC*) is the ratio of a particle's density to the particle's total density in the box along a given average direction near a distance of *r*. It analyses the variation of the concentration of individual particles in the system and is calculated as shown in (4).

4 $$\rho_{r} = \frac{{\rho_{i} }}{{\rho_{{\text{total }}} }}$$

*ρ*_*r*_ is the relative concentration of a particle in the region *r*; *ρ*_*r*_ is the number density of the particle in the region *r*, and *ρ*_*tota*l_ is the total number density of the particle in the whole system. Relative concentration distribution curves along the z-axis of the coal/surfactant/water system^45^ were analyzed using the Forcite module.

#### Bituminous coal-water interaction energy

The allocation of water molecules under the whole adsorption system is the result of the joint action of water, bituminous coal and different types of surfactants, so it is also very important to understand the interaction mechanism between bituminous coal molecules and water molecules. The change of adsorption properties of modified coal and water by different surfactants can be effectively reflected by calculating the interaction energy of bituminous coal and water, and its value reflects the strength of the interaction energy between modified coal molecules and water molecules. The calculation formula is as follows.

5 $$Int_{c + w} = (E_{total} - E_{c + s} - E_{s + w} - E_{c} - E_{w} + E_{s} + E_{c + w} )/2$$

In Eq. (5), *Int*_*c*+*w*_ is the magnitude of the interaction energy between bituminous coal molecules and water molecules; *E*_*total*_ is the magnitude of the total energy of the whole coal-surfactant-water system; *E*_*c*+*s*_ is the magnitude of the energy of the coal and the surfactant; *E*_*s*+*w*_ is the energy of the surfactant and the water; *E*_*c*+*w*_ is the energy of the coal and the water; and *E*_*c*_, *E*_*w*_ and *E*_*s*_ are the energies of the coal, water and surfactant, respectively.

#### Radial distribution function

The radial distribution function (*RDF*), which is the probability of finding ions around a particle centered on that particle in a given space, is also understood in software simulations as the ratio of the regional density of a periodic bounding box to the global density. For a crystalline structure, where the position of each atom is fixed and immobile, it is an ordered structure, so it has a radial distribution function with long-range peaks, and for an amorphous structure, it generally has a short-range peak distribution.

The radial distribution function analyses, to some extent, the interactions and mutual positions of the atoms, and its radial distribution function g(*r*) for particle B around particle A is expressed as in Eq. (6).

6 $$G_{A - B} (r) = \frac{1}{{4\pi \rho_{B} r^{2} }} \cdot \frac{{dN_{A - B} }}{dr}$$

where *dN*_*A-B*_ is the average number of B particles in the range from *r* to *r* + d*r*, *ρ*_*B*_ is the density of B particles, and *r* is the distance between A and B particles. Where the value of the largest radial distance, *r*_*max*_, cannot be more than half the minimum side length of the box.

The total number of particles in the *r* region, the coordination number (*CN*), is an important indicator of the strength of the intermolecular interactions. The calculation formula is given in (7).

7 $${\mathbf{CN}}({\mathbf{r}}) = \int_{0}^{r} 4 \pi {\mathbf{r}}^{2} \rho {\mathbf{g}}({\mathbf{r}}){\text{d}}{\mathbf{r}}$$

#### The mean square displacement curve

The mean square displacement (*MSD*), which is a measure of the deviation of the position of a particle relative to a reference position as it moves over time, further explains the extent of the effect on the movement of water molecules caused by the addition of surfactant^46^. The calculation of the mean square displacement is shown in Eq. (8).

8 $${\text{MSD}} = \frac{1}{N}\sum\limits_{i = 1}^{N} {\left( {r_{i} (t) - r_{i} (0)} \right)^{2} }$$

where *N* is the total number of diffusing molecules and *r*_*i*_(*t*) and *r*_*i*_(*0*) are the positions of the diffusing molecules.

### Simulation settings

The simulation was performed using Materials Studio 2020 for simulation studies. The setup parameters for the ensemble optimization are as follows: the force field is selected as COMPASS III, the electrostatic force is calculated by the Ewald method, the calculation accuracy is selected as Ultra-fine, the maximum number of iteration steps is set to 5000, the energy is set to 2.0e-5 kcal/mol, and the force is set to 0.001 kcal/mol/Å. The setup parameters for annealing optimization are as follows: annealing is performed at 300.0 to 600.0 K, the annealing cycle is set to 8 times, the coefficient is selected as NVT, the number of steps is set to 16,000 annealing optimization steps, and the energy deviation is set to 50,000 kcal/mol. The parameter settings of the Dynamic Optimization module are as follows: Initial velocities are set to Random, Force Field is set to COMPASS III, and the total simulation time is set to 400 ps.

A simulation system was composed of boxes made up of three amorphous modules, Wiser coal, surfactant, and water, by building Build Layers. The bottom two-thirds of the coal polymerization molecules were fixed to save computational time, and it has been shown that this practice has little effect on the final results^30,47^. The three assembled systems were subjected to kinetic calculations with the setup parameters shown in Table 2.

**Table 2:** Set the relevant parameters.

Dynamics options	Parameter
Ensemble	NVT
Temperature	298.0 K
Control method	Nose
Timestep	1.00 fs
Duration	400 ps
Forcefield	COMPASSIII
Summation method	Ewald
%1.%2 Accuracy	%1.%2 al/mol

### Molecular dynamics (MD) simulation of the system

Since the potential energy surface of macromolecules has local energy minima and global energy minima, and the variation is relatively complex, the global energy minima can be found by MD simulation in Materials Studio software, and molecular dynamics (MD) simulation is the most widely used computational method for calculating large systems in the present era.

Molecular dynamics analyses of the three surfactants and Wiser coal were carried out using the Forcite module of MS software to find low-energy configurations of the molecules on the surface. Geometry optimization and annealing calculations are carried out on individual molecules of the surfactants and coal to bring the model energy states to a steady state.

**Figure 2:**
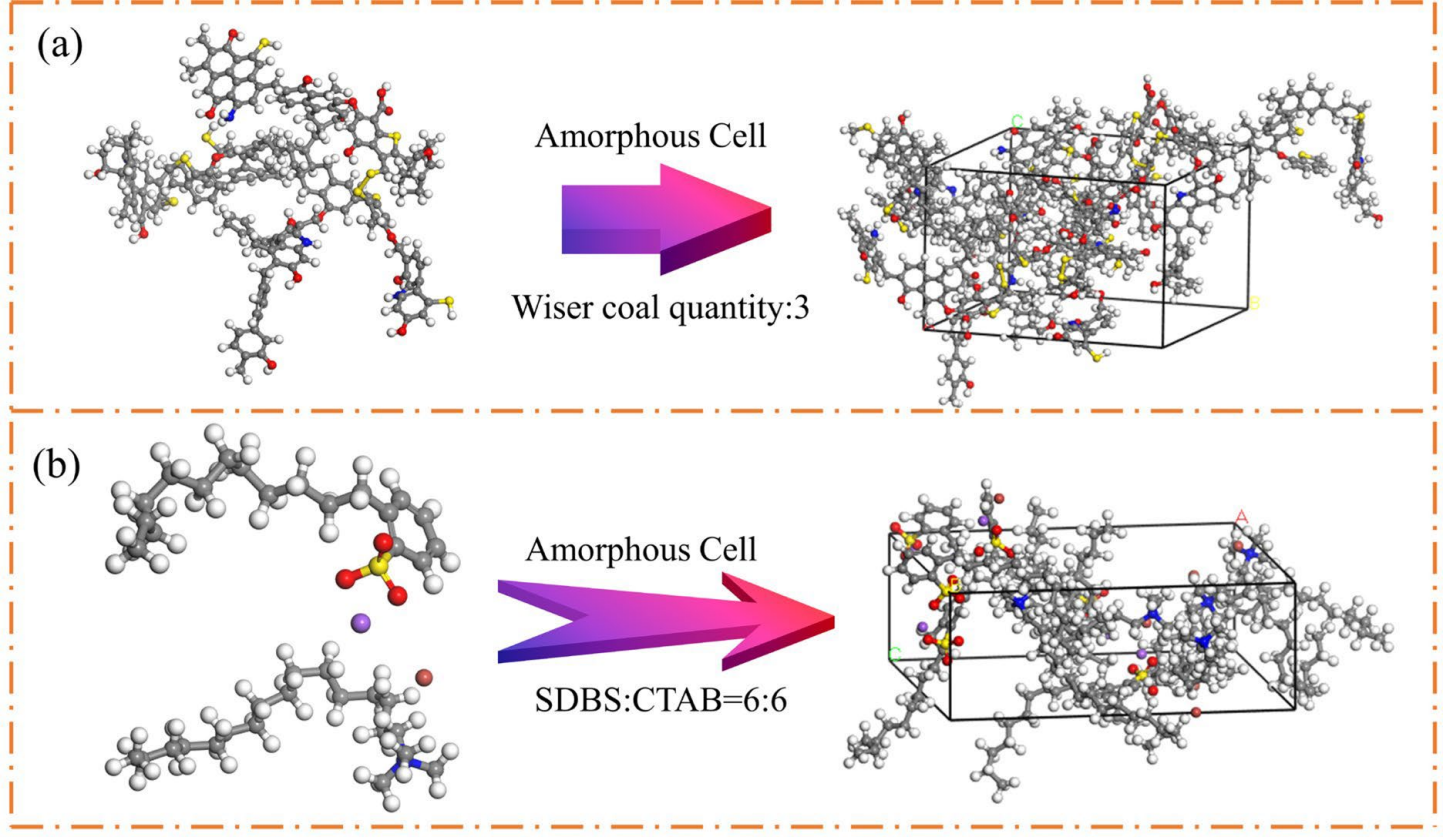
(**a**) Wiser coal aggregate state model, (**b**) aggregate state model formed by the composite of two surfactants.

**Figure 3:**
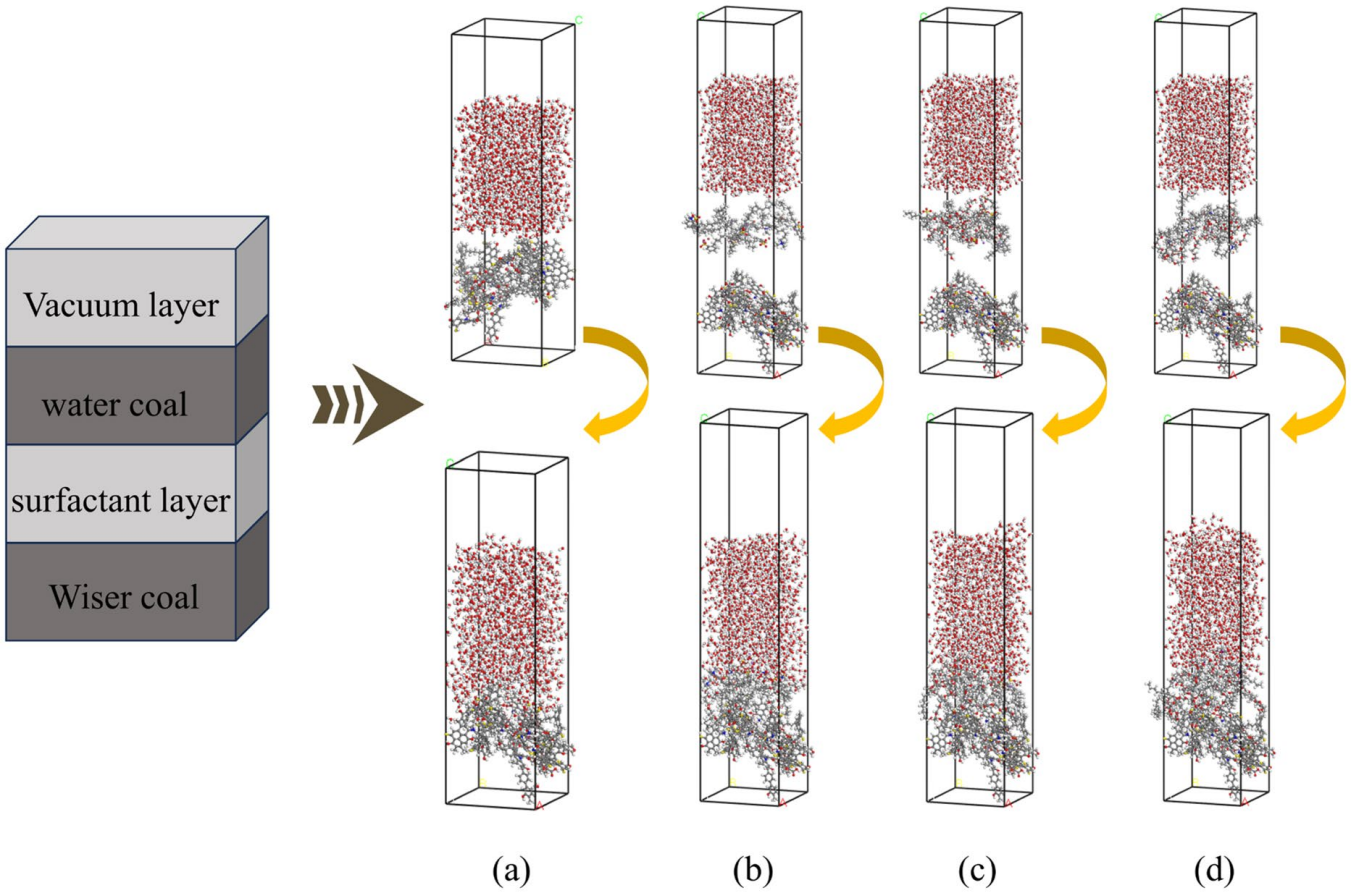
Coal-compound surfactant-water three-phase model. (**a**) no surfactant, (**b**) SDCT, (**c**) SDAE, (**d**) CTAE.

As shown in Fig. 2, the optimized surfactant molecules and the molecular structure of Wiser bituminous coal were used to establish the spatial configurations using the Amorphous Cell, in which the anionic-cationic (SDBS-CTAB), anionic-nonionic (SDBC-AEO9) and cationic-nonionic (CTAB-AEO9) surfactants were compounded two-by-two. The total number of active ingredients was 12 molecules in a 1:1 ratio; a three-dimensional aggregation configuration containing three Wiser bituminous coal macromolecules was established. The size of the simulation system and the schematic diagram are shown in Table 3 and Fig. 3.

**Table 3:** The size of the coal-compound surfactant-water composition model.

model	N_c_	N_s_	N_w_	Cube size (Å)
No surfactant	3	0	1000	27.30 × 27.30 × 94.56
SDCT	3	6:6	1000	27.30 × 27.30 × 119.68
SDAE	3	6:6	1000	27.30 × 27.30 × 121.26
CTAE	3	6:6	1000	27.30 × 27.30 × 123.12

SDCT(SDBS-CTAB), SDAE(SDBS-AEO9), CTAE(CTAB-AEO9); N_c_, N_s_, and N_w_ are the quantities of coal, surfactant, and water, respectively.

## Discussion and results

### Analysis of molecular electrostatic potential graphs

The maximum positive and maximum negative potentials of the transformed molecule are shown in Table 4. The positive and negative electrostatic potentials reflect, to some extent, the electrical distribution of the molecule, which is presented according to the pattern of "blue-white-red", showing that the red region is positively charged, which is more difficult to lose electrons and more likely to accept electrons to form hydrogen bonds, the blue region is negatively charged and more likely to provide electrons to the positively charged region to form hydrogen bonds, while the white region is electrically neutral and the structure will be relatively stable^48^. The shade of color indicates the magnitude of the electrostatic potential.

**Table 4:** Maximum electrostatic potential (× 10^–19^ J).

Type	Maximum positive potential	Maximum negative potential
coal	4.915	− 3.718
H_2_O	3.992	− 2.854
SDBS	10.608	− 3.581
CTAB	3.283	− 4.544
AEO9	3.810	− 3.435

**Figure 4:**
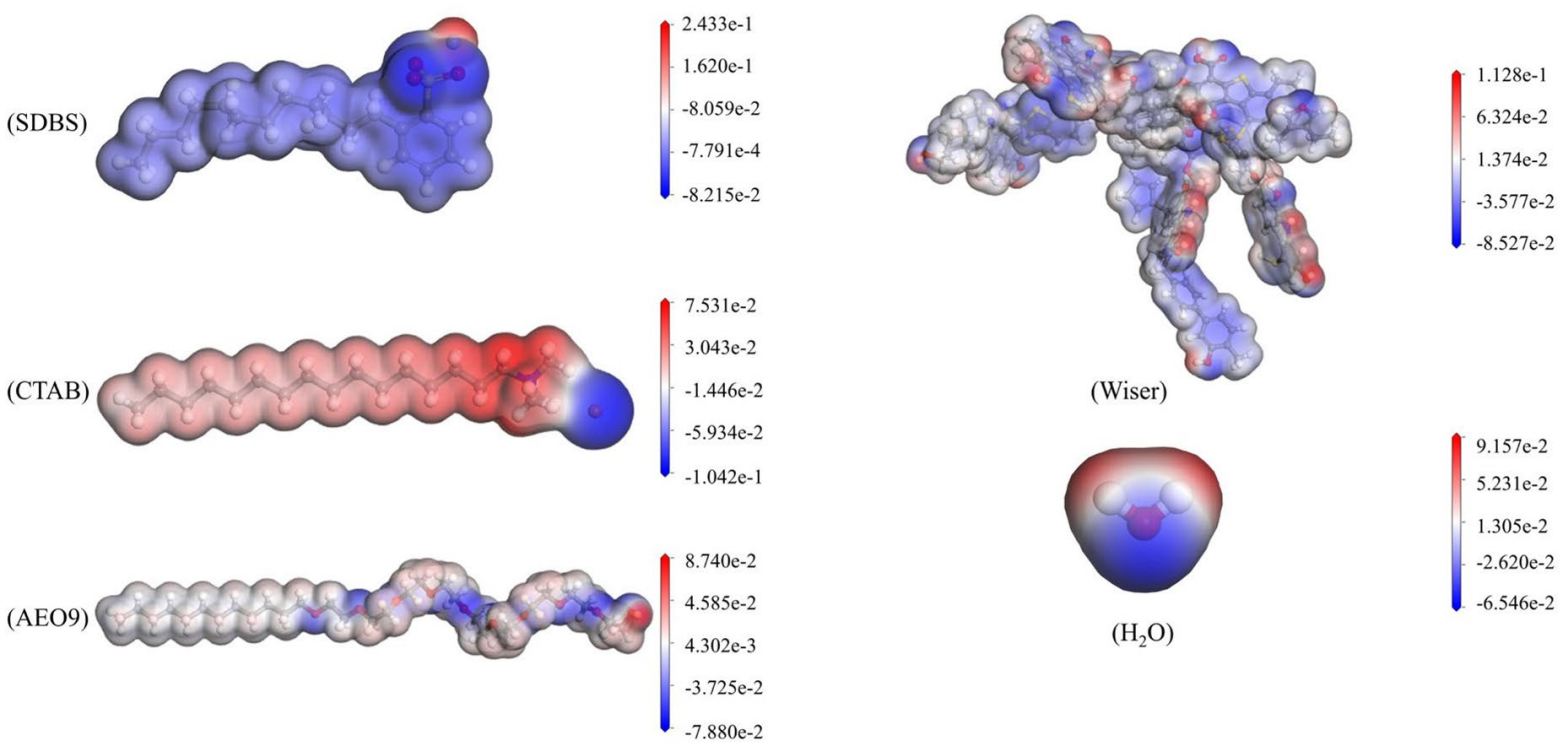
Electrostatic potential profiles of surfactants, coal, and water molecules.

Figure 4 shows the distribution area of the electrostatic potential of coal molecules, water molecules, and surfactant molecules. From Fig. 4, it can be seen that the anionic surfactant (SDBS) has good adsorption properties, which can form a monomolecular membrane and improve the contact area between carbon and bubbles. Most of the area in SDBS is negative potential, and the maximum electrostatic potential is concentrated at the site of sulfonate ions (RSO_3_), and the repulsion between them makes the electric field strength of the area higher; while the minimum potential is in the vicinity of alkyl chains (-R), where the electric field strength is weaker. The strong interaction between the negative charge of the carbon dust and the sulfonate ions is the anionic group away from the surface of the carbon dust, and the alkyl chain adsorbed on the surface of the carbon dust^49^.

In contrast, the cationic type (CTAB) dissociates in water with a positive charge, changing the nature of the charge on the carbon surface. For the CTAB surfactant, the maximum electrostatic potential occurs around the quaternary ammonium cation (NR + 4). For the non-ionic surfactant AEO-9, the maximum electrostatic potential is mainly distributed around ethylene oxide (–CH_2_CH_2_O–)^50^, and the static potential gradually becomes neutral as the distance of the carbon atoms on the alkyl chain from the head increases and the force between the surfactant and the coal molecules is mainly van der Waals force. The surfactant molecules are aggregated around the coal molecules^51^. This type of surfactant can regulate the surface tension of the carbon and stabilize bubble formation.

The distribution of the electrostatic potential of Wiser bituminous coal is mainly concentrated near the functional groups it carries, such as hydroxyl, carboxyl, sulfur, ether, ammonia, and a number of other oxygen-, sulfur- and nitrogen-containing functional groups. The electrostatic potential tends to be neutral in the region near the benzene ring due to the delocalization of the π electron cloud around the benzene ring.

Normally, water molecules attract each other under the action of positive and negative potentials, and the maximum positive electrostatic potential of water is 3.992 × 10^–19^ J and the maximum negative electrostatic potential is − 2.854 × 10^–19^ J. The surfactant has a hydrophilic head and a hydrophobic tail. Suppose the coal molecules or those modified by the surfactant have a maximum electrostatic potential greater than that of water. In that case, its hydrophilic region will attract water molecules with opposite charges to form hydrogen bonds, forming a bridge between water and coal and changing the hydrophilicity of the coal.

### Orbital distribution characteristics

It has been shown that reactions between ground-state molecules occur through the most efficient overlap between LUMO and HOMO. The difference in energy levels between the two (LUMO–HOMO) band gaps ΔE is used to measure the degree of reactivity; the smaller the difference, the easier it is to excite; the more significant the difference, the more stable the molecule. The molecular stability of surfactants is positively correlated with the wetting effect of carbon^52^.

**Figure 5:**
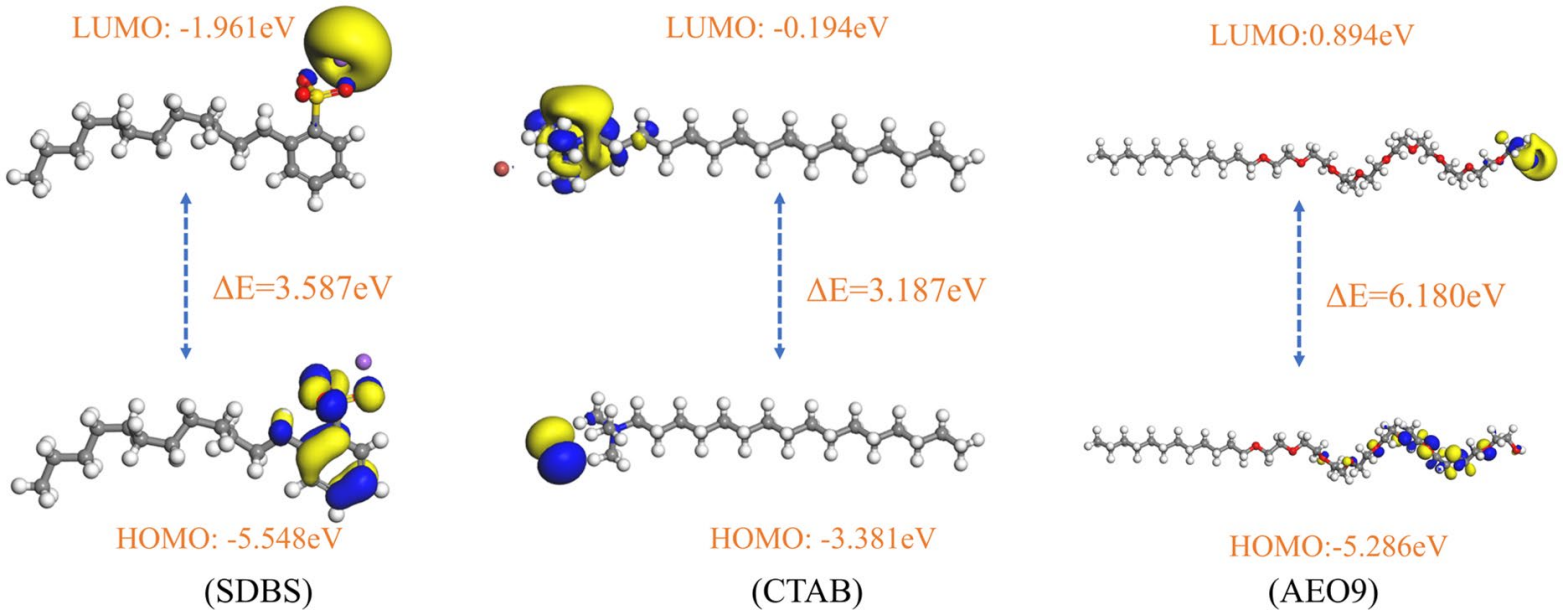
Track diagram of the front of each surfactant.

Figure 5 and Table 5 show the size of the LUMO, HOMO, and energy level difference for different types of surfactants. From Fig. 5, it can be seen that the frontier orbital energy difference of the three surfactants AEO-9, SDBS, and CTAB are 6.180 eV, 3.587 eV, and 3.187 eV, respectively, and their values decrease gradually. The stability of AEO-9, SDBS, and CTAB also decreases sequentially. The energy level difference (ΔE = 6. 180 eV) of the non-ionic surfactant AEO-9 is the largest, which is due to the fact that the non-ionic surfactant AEO-9 becomes electrically neutral and will achieve stable adsorption at the coal-water interface. In contrast, the cationic surfactant CTAB has the smallest difference in frontier orbital energy, which can improve coal's hydrophobic properties.

**Table 5:** LUMO–HOMO energy band gap *ΔE* (eV).

surfactant	E_HOMO_	E_LUMO_	ΔE
SDBS	− 5.548	− 1.961	3.587
CTAB	− 3.381	− 0.194	3.187
AEO-9	− 5.286	0.894	6.180

### Relative concentration distribution along the Z-axis

**Figure 6:**
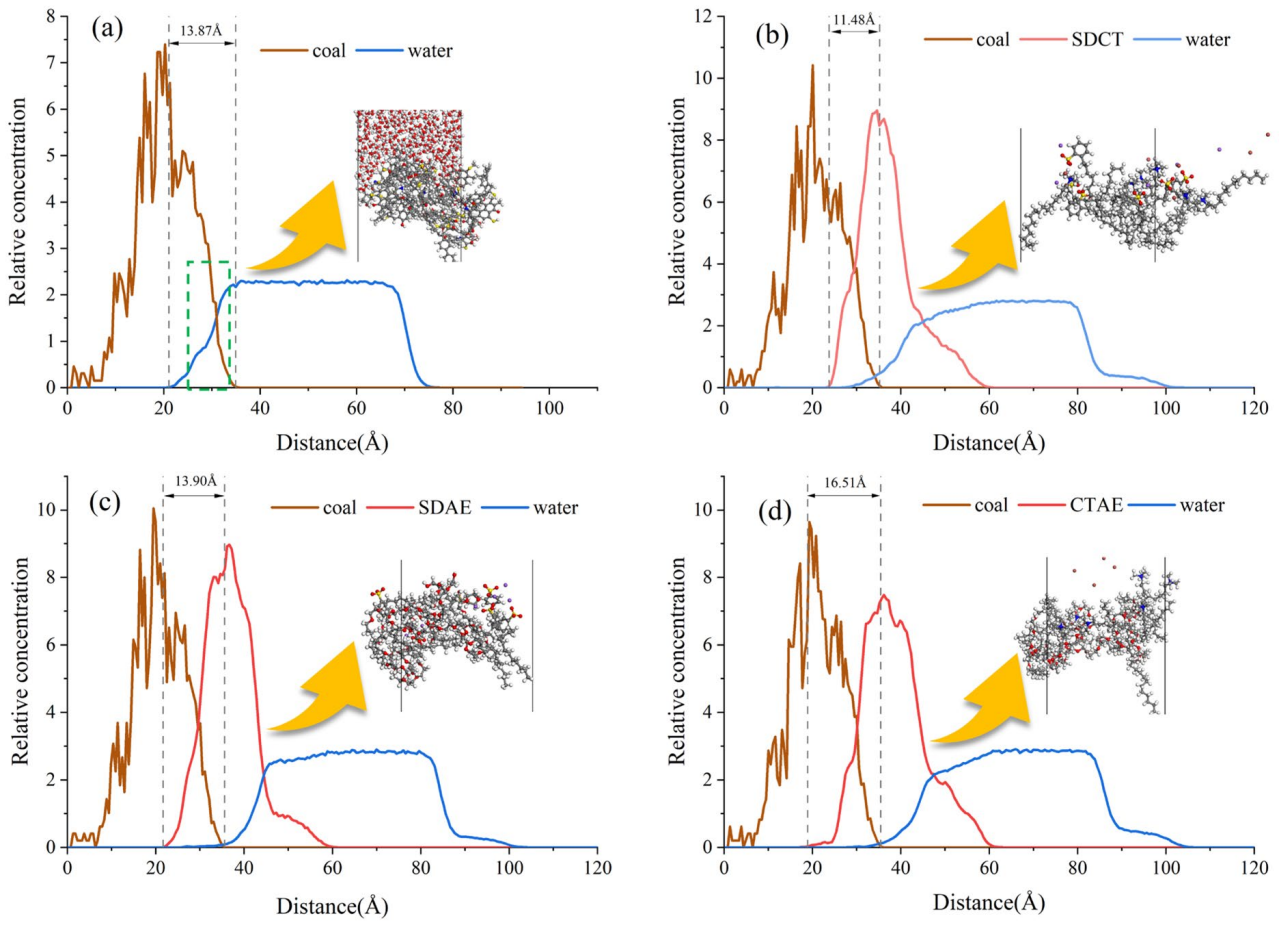
(**a**–**d**) are the relative concentration distributions of coal/water, coal/SDCT/water, coal/SDAE/water, and coal/CTAE/water along the Z-axis, respectively.

Figure 6 shows the relative concentration distribution of different bituminous coal/surfactant/water along the Z-axis. From Fig. 6, it can be seen that the peak positions in the curves represent the places where the concentration of molecules, atoms, or groups is concentrated. Since the lower two-thirds of the coal molecular polymer was fixed in advance before molecular dynamics was performed, the position of the coal molecules did not change too much during the adsorption process. It remained stable at a position around 0–35.26 Å.

In the coal/water molecule system, there is a partial overlap of the relative concentration curves of the two. On the one hand, due to the roughness of the surface of the coal molecules, there are a variety of uneven positions, and the water molecules are filled into the pores of the coal molecules. On the other hand, due to the intermolecular forces between the coal and water molecules,

In the coal/surfactant/water system, with the addition of compound surfactant, the surfactant molecules fill the pores in the coal molecules, which is reflected in the figure as the overlap of the curves of the coal molecules and water molecules is significantly reduced, and the surfactant isolates the contact between coal and water. In the adsorption process, the hydrophilic structure of each surfactant, such as sulfonate, quaternary ammonium cation, and other hydrophilic structures, will gradually turn to the water phase, and it is easy to have hydrogen bonding with the water molecules; the ethylene oxide group of the non-ionic surfactant will gradually shift to the water molecules, and the alkyl chain in the tail will turn to the surface of the coal molecules.

By measuring the size of the overlapping portion of the relative concentration curves of the coal molecules and surfactant molecules, which is SDCT (11.48 Å) < SDAE (13.90 Å) < CTAE (16.51 Å), there is a strong synergistic effect between the compounded surfactant CTAB and the coal molecules, which improves the adhesion of the compounded surfactant on the surface of the coal by reducing the surface tension^46^. Therefore, it can be judged that the modified coal with surfactant compounded by CTAE has good hydrophobicity.

### Interaction energy analysis

**Figure 7:**
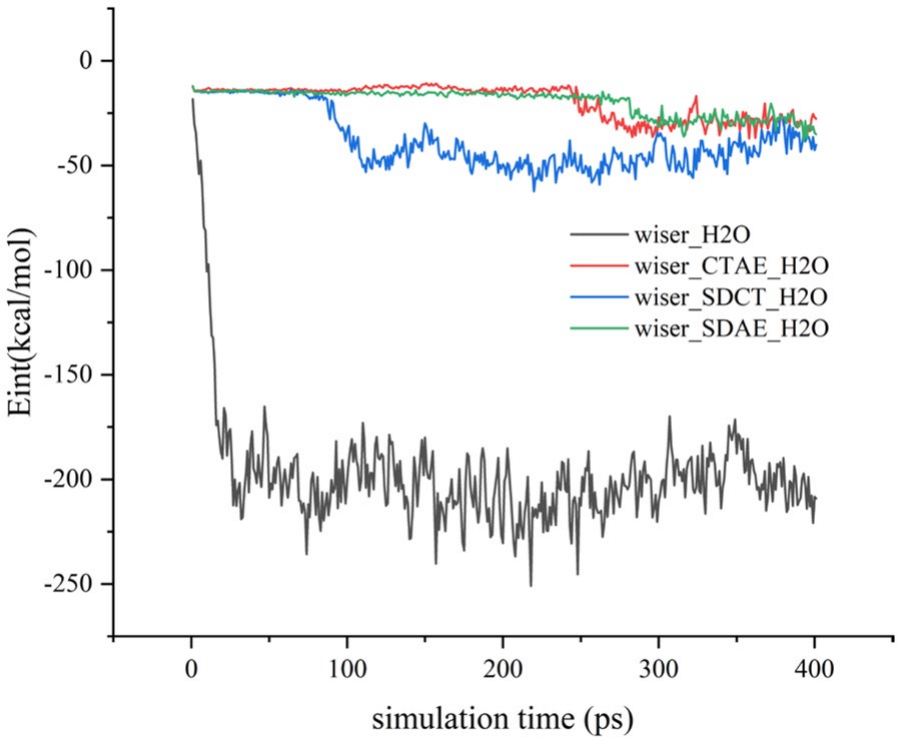
Coal-water intermolecular interaction energy curve over time.

Table 6 calculates the magnitude of the intermolecular, van der Waals, and electrostatic interaction energies between coal and water in different systems. The calculated interaction energy magnitudes are all negative, indicating that the adsorption process is spontaneous. Figure 7 shows the variation curve of the interaction energy between coal and water molecules with time. From Fig. 7, it can be seen that the interaction energies between water molecules and bituminous coal molecules in SDCT, SDAE, and CTAE systems were − 40.169 kcal/mol, − 35.085 kcal/mol, and − 27.713 kcal/mol, respectively, and the magnitude of the absolute value of the interaction energy between coal and water has decreased significantly, as has the interaction force between modified coal and water. In addition, the interaction force between modified coal and water decreased. The absolute values of intermolecular interaction energies of different systems were ranked as No surfactant > SDCT > SDAE > CTAE, indicating that coal_CTAE_H2O has better hydrophobicity in this system.

**Table 6:** Energy in each simulation system (unit: kcal/mol).

system	Interaction energy	van der waals	Electrostatic
No surfactants	− 209.159	− 79.585	− 129.574
SDCT	− 40.169	− 9.723	− 30.446
SDAE	− 35.085	− 11.311	− 23.774
CTAE	− 27.713	− 8.021	− 19.692

The magnitudes of the van der Waals and electrostatic energies were calculated, and by comparing Table 6, the magnitude of the electrostatic energy is generally more prominent than the van der Waals energy, and the electrostatic force plays a dominant role in the adsorption process.

### Radial distribution function

**Figure 8:**
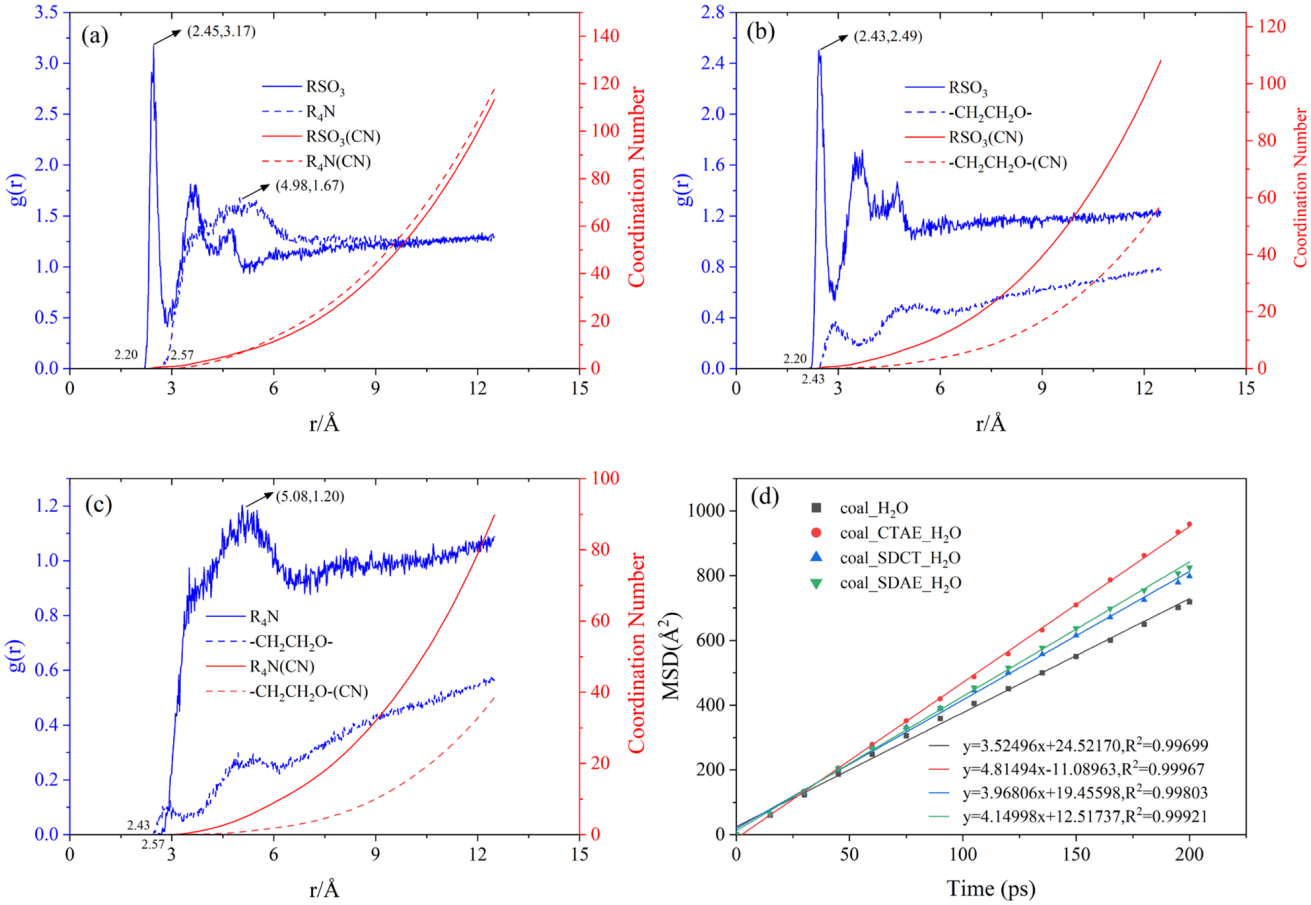
(**a**–**c**) are the radial distribution functions and coordination numbers of SDCT, SDAE, and CTAE surfactants and water molecules, respectively, (**d**) Mean square displacement curves of water molecules in different systems and their fitting straight lines.

By analyzing the electrostatic potential and orbital energy difference of each type of surfactant above, the site of maximum electrostatic potential of the surfactant compounded in each system was analyzed in terms of radial distribution with the oxygen atoms in water. The sulfonic acid group (RSO_3_) of the anionic surfactant; the quaternary ammonium cation (R_4_N) in the cationic surfactant and the ethylene oxide (–CH_2_CH_2_O–) in the nonionic surfactant were employed as Particle A, while the oxygen atoms in the water were utilized as Particle B. The results of the analyses are shown in Fig. 8a–c.

From Fig. 8a–c, it can be seen that all three simulation systems have a tendency towards g(*r*) = 1 with increasing distance, i.e. they tend towards the overall density of the box. When anionic/cationic surfactants are present in the system, there are different degrees of peaks. The peak coordinates of the radial distribution functions of the sulfonic acid group RSO3 and the quaternary ammonium cation R4N in the SDCT system are (2.45, 3.17) and (4.98, 1.67), respectively, and the peaks on the curve of the sulfonic acid group are higher. The peaks are closer to each other than those of the quaternary ammonium cation, indicating that the intermolecular forces between the surfactant and water are stronger. The surface tension at the interface is lower. In the system with non-ionic surfactant, it was found that the radial distribution curve was always on an upward trend, and there was no peak, indicating that the interaction with water was very small and even played a repulsive role.

When g(*r*) = 0, it shows that there is a repulsive force between surfactant molecules and water molecules with a distance of *r*. The smaller the repulsive force distance, the greater the intermolecular interaction force. Comparing the repulsive force distances of the three systems sulfonate (2.20) < Ethylene oxide (2.43) < Quaternary ammonium cation (2.57), the strength of the intermolecular interactions from largest to smallest is Sulfonate (RSO_3_) > Ethylene oxide (–CH_2_CH_2_O–) > Quaternary ammonium cation (R_4_N).

From the coordination number point of view, its value CTAE < SDAE < SDCT, comprehensive analysis, according to CTAE compound surfactant modified coal hydrophobic performance, will be better.

### Diffusion behavior

In the coal/surfactant/water system, the variation in the degree of diffusion of water is reflected by the mean square displacement (MSD) and the diffusion coefficient (D). The MSD curves of each simulated system and their respective fitted linear distributions were taken as shown in Fig. 8d. The following equation shows the formula for the molecular diffusion coefficient (D).

9 $$D = \frac{1}{6N}\mathop {\lim }\limits_{t \to \infty } \frac{{\text{d}}}{{{\text{d}}t}}\sum\limits_{1}^{N} {\left[ {r_{i} (t) - r_{i} (0)} \right]^{2} }$$

That is, the diffusion coefficient is 1/6 of the slope of the MSD scatterplot shape^53^. From Fig. 8(d), it can be seen that the diffusion coefficients of coal_H_2_O, coal_SDCT_H_2_O, coal_SDAE_H_2_O, coal_CTAE_H_2_O are 5.87 × 10^–21^ m^2^/ps, 6.61 × 10^–21^ m^2^/ps, 6.92 × 10^–21^ m^2^/ps, 8.02 × 10^–21^ m^2^/ps in increasing order. It shows that by adding a compound surfactant, the diffusion coefficients of water are all larger than the value of a surfactant-free structure, which improves the hydrophobicity of coal and increases the water migration rate, which is favorable for coal flotation.

In conclusion, the binary surfactants CTAB and AEO-9 were the most effective in modifying coal, resulting in the highest hydrophobicity. This finding aligns with the results of Zhang et al.^39^, who demonstrated that AEO-9/SDS and AEO-9/CTAB mixtures exhibited minimal synergistic effects in measuring the effect of surface tension reduction. This corroborates the veracity of the results of the present study.

## Conclusion

The following are the conclusions drawn from the simulations conducted to investigate the magnitude of the activity of different types of surfactants undergoing adsorption at room temperature, to predict the occurrence of electrophilic and nucleophilic reaction sites, and to analyze in detail the relative concentration distributions, interaction energies, radial distribution curves, and diffusion coefficients in the bituminous coal/ binary surfactant/ water system.

1. The maximum electrostatic potentials of cationic/anionic/nonionic surfactants (CTAB/SDBS/AEO-9) were determined by electrostatic potential analysis to be 4.544 × 10^–19^ J, 10.608 × 10^–19^ J, and 3.810 × 10^–19^ J. The values were distributed around the quaternary ammonium cations (NR + 4), sulfonate ions (RSO_3_), and ethylene oxide (-CH_2_CH_2_O-) which are surrounded by groups with high electronegativity by O and S. These groups regulate the surface tension of the coal.
2. Molecules with a smaller frontier orbital energy difference *ΔE* exhibit higher activity, which facilitates their interaction with coal and enhances its hydrophobicity. The magnitude of *ΔE* for the three surfactants (SDBS/CTAB/AEO-9) is 3.587 eV, 3.187 eV, and 6.180 eV, respectively. The order is as follows: The order of the surfactants (SDBS, CTAB, and AEO-9) is AEO-9 > SDBS > CTAB. Therefore, CTAB is the most effective trapping agent molecule.
3. The interaction energy between water molecules and bituminous coal molecules in different systems is all negative, indicating that the adsorption process is spontaneous. Furthermore, the absolute values are all smaller than those of non-surfactants (-209.159 kcal/mol), suggesting that all of them can improve the hydrophobicity of coal.
4. Analyzing the kinetic behavior between surfactants and water molecules, it can be found that the magnitude of the force between hydrophilic groups of surfactants and water molecules is in the following order, sulfonic acid group (RSO_3_) > ethylene oxide (-CH_2_CH_2_O-) > quaternary ammonium cation (R_4_N), and the magnitude of the diffusion coefficients of the different systems are No surfactants (5.87 × 10^–21^ m^2^/ps) < SDCT (6.61 × 10^–21^ m^2^/ps) < SDAE (6.92 × 10^–21^ m^2^/ps) < CTAE (8.02 × 10^–21^ m^2^/ps), and it can be seen from the above analyses that in the coal/CTAE/water system, the modification of coal hydrophobicity by CTAE complex surfactant is better, and the flotation performance of coal is better than the other two.

### Supplementary Information

Supplementary Information.

## Data Availability

Data is provided within the supplementary information files.
